# Reevaluating Adiponectin’s impact on obesity hypertension: a Chinese case-control study

**DOI:** 10.1186/s12872-024-03865-4

**Published:** 2024-04-13

**Authors:** Ou Wu, Xi Lu, Jianhang Leng, Xingyu Zhang, Wei Liu, Fenfang Yang, Hu Zhang, Jiajia Li, Saber Khederzadeh, Xiaodong Liu, Chengda Yuan

**Affiliations:** 1 Shulan International Medical College, Zhejiang Shuren University, Hangzhou, Zhejiang People’s Republic of China; 2 Zhejiang Chinese Medical University, Hangzhou, Zhejiang People’s Republic of China; 3 Hangzhou Vocational and Technical College, Hangzhou, Zhejiang People’s Republic of China; 4 Department of Central Laboratory/Medical Examination Center of Hangzhou, The Frist People’s Hospital of Hangzhou, Hangzhou, Zhejiang People’s Republic of China; 5 Thomas E. Starzl Transplantation Institute, University of Pittsburgh Medical Center, Pittsburgh, PA USA; 6 JFIntelligent Healthcare Technology Co., Ltd Building No.5-7, No.699 Tianxiang Avenue, Hi-Tech Zone, Nanchang, Jiangxi Province People’s Republic of China; 7 Department of Thoracic Surgery, Sir Run Run Shaw Hospital Affiliated with Medical College of Zhejiang University, Hangzhou, Zhejiang People’s Republic of China; 8 Department of Central Laboratory, The First Affiliated Hospital of Anhui Medical University, Hefei, Anhui People’s Republic of China; 9 Westlake Laboratory of Life Sciences and Biomedicine, Hangzhou Zhejiang, People’s Republic of China; 10 Institute of Basic Medical Sciences, Westlake Institute for Advanced Study, Hangzhou Zhejiang, People’s Republic of China; 11 Hangzhou Center for Disease Control and Prevention, Hangzhou, Zhejiang People’s Republic of China; 12 Department of Dermatology, Hangzhou Hospital of Traditional Chinese Medicine, Hangzhou, Zhejiang People’s Republic of China

**Keywords:** Adiponectin, Obesity, Hypertension, Blood pressure, Visceral adiposity index, Alkaline phosphatase

## Abstract

**Background:**

Obesity and hypertension are major risk factors for cardiovascular diseases that affect millions of people worldwide. Both conditions are associated with chronic low-grade inflammation, which is mediated by adipokines such as adiponectin. Adiponectin is the most abundant adipokine that has a beneficial impact on metabolic and vascular biology, while high serum concentrations are associated with some syndromes. This “adiponectin paradox” still needs to be clarified in obesity-associated hypertension. The aim of this study was to investigate how adiponectin affects blood pressure, inflammation, and metabolic function in obesity hypertension using a Chinese adult case-control study.

**Methods:**

A case-control study that had finished recruiting 153 subjects divided as four characteristic groups. Adiponectin serum levels were tested by ELISA in these subjects among these four characteristic Chinese adult physical examination groups. Waist circumference (WC), body mass index (BMI), systolic blood pressure (SB), diastolic blood pressure (DB), and other clinical laboratory data were collected. Analyzation of correlations between the research index and differences between groups was done by SPSS.

**Results:**

Serum adiponectin levels in the| normal healthy group (NH group) were significantly higher than those in the newly diagnosed untreated just-obesity group (JO group), and negatively correlated with the visceral adiposity index. With multiple linear egression analysis, it was found that, for serum adiponectin, gender, serum albumin (ALB), alanine aminotransferase (ALT) and high-density lipoprotein cholesterol (HDLC) were the significant independent correlates, and for SB, age and HDLC were the significant independent correlates, and for DB, alkaline phosphatase (ALP) was the significant independent correlate. The other variables did not reach significance in the model.

**Conclusions:**

Our study reveals that adiponectin’s role in obesity-hypertension is multifaceted and is influenced by the systemic metabolic homeostasis signaling axis. In obesity-related hypertension, compensatory effects, adiponectin resistance, and reduced adiponectin clearance from impaired kidneys and liver all contribute to the “adiponectin paradox”.

## Introduction

Obesity, having an inexorable rise with over 650 million obese worldwide [1], is globally a significant public health challenge and accounts for 65–78% of cases of primary hypertension [2]. The pathways through which obesity causes hypertension are complicated, including sympathetic nervous system overactivation, stimulation of the renin-angiotensin-aldosterone system, alterations in adipose-derived cytokines, insulin resistance, and structural and functional renal changes [2]. Adipose tissue is one of the largest endocrine organs in the body playing multiple intricate roles including secretion of a number of biologically adipokines, of which the most abundant is adiponectin [1].

Adiponectin, a 28-kDa protein adipocytokine, mainly produced and secreted into the circulation by lean adipocytes [3, 4], has been very widely studied, over the past 25 years, since mid-1990s [5]. The primary function of adiponectin is the regulation of carbohydrate and lipid metabolism [6]. However, the full extent of its biological action remains to be elucidated, with a variety of effects on different cell and tissue types [3, 7]. Adiponectin is initially considered a guardian angel adipocytokine owing to its protective functions against various disease states associated with obesity, such as immune modulatory, insulin­sensitizing, antidiabetic, anti­obesogenic, anti­inflammatory, antiatherogenic, anti-fibrotic, cardio­ and neuroprotective properties [3, 4, 7]. Adeno-viral over-expression of adiponectin in obese KKAy/a mice resulted in reduced blood pressure and reversed salt-induced hypertension [8]. Contrariwise to its protective effects against various pathological events in different cell types, adiponectin may have links to several systemic diseases and malignancies [1]. Accumulating reports of results of several meta-analyses demonstrated that elevated serum levels of both total and High Molecular Weight (HMW) adiponectin have been positively associated with both cardiovascular and, what is quite surprising and confusing, even with the all-cause mortality rate in the population above 65 years of age [9]. The biology underlying this paradox is still being studied [9].

This “adiponectin paradox” is remained, when coming to the role of adiponectin in hypertension. On one hand, compared to the patients with essential hypertension, adiponectin plasma levels were found to be significantly higher in the normotensive subjects [1, 10]. And lower adiponectin levels were associated higher blood pressure or with incident hypertension [11–14]. These phenomena may be due to their protecting role on vascular functions through improving the functions of macrophages and endothelial cells. These mechanisms may include attenuating the phenotype of macrophages M1 and to promote the phenotype of macrophages M2 and preventing endothelial dysfunction through enhancing endothelial nitric oxide synthase (eNOS) activity and nitric oxide (NO·) production via adiponectin receptors AdipoR1/R2-AMPK (Adenosine 5′-monophosphate (AMP)-activated protein kinase)-endothelial signaling and cyclooxygenase-2 (COX-2) expression and prostaglandin I2 (PGI2) production by means of calreticulin/CD91-dependent Akt (protein kinase B) signaling [15]. And adiponectin also can lower blood pressure by decreasing renal sympathetic nervous system activity through its short-lived action on brain in adiponectin knock-out mice [15–21].

On the other hand, chronic intracerebroventricular infusion of adiponectin did not alter blood pressure in normotensive or hypertensive rats [10, 22]. And Sprague-Dawley rats fed a high salt diet exhibited hypertension associated with elevated levels of adiponectin, suggesting that adiponectin does not play a protective role against salt-induced hypertension [20, 23]. Adiponectin KO mice developed hypertension without insulin resistance when maintained on a high-salt diet, indicating that hypoadiponectinemia, per se, is not sufficient for the development of hypertension but contributes to its development under insulin resistance and/or salt overload [8]. In normotensives and untreated hypertensives with normal kidney function, adiponectin is not associated with blood pressure even after adjustment for many risk factors [24]. Several longitudinal studies offer mixed insights into adiponectin’s influence on metabolic diseases like hypertension, with some linking high levels to reduced metabolic syndrome risk and others to increased mortality and no protection against hypertension [11, 13, 25–29]. These studies highlight the complexity of adiponectin’s role in metabolic health and the need for further research to clarify its impact. This study aimed to provide more clues for distinguishing the antinomy of adiponectin in obesity hypertension.

## Data and methods

### Study design and participants

This was a case-control study involving 153 Chinese adults (aged 40.78 ± 11.77 years; 119 men, 34 women) who underwent annual physical examinations in a hospital from September 2012 to July 2014 [30]. The participants were divided into four groups based on their body mass index (BMI) and blood pressure: the normal healthy (NH) group, the newly diagnosed untreated just-obesity (JO) group, the newly diagnosed untreated just-hypertension (JH) group, and the newly diagnosed untreated obesity-hypertension (OH) group. The inclusion and exclusion criteria for each group were set as previously reported [30–34] and are shown in Table 1. The number of subjects included in each group is also shown in this table. BMI was obtained by dividing the body weight by the square of the respective subject’s height (weight/height^2^ (kg/m^2^)) .

**Table 1:** Selection criteria for subjects and the included subject numbers in this case-control study

Group	Inclusion Criteria	Exclusion Criteria
NH	• People without a clinically serious ailment, such as obesity, hypertension, or another condition	• Not meet the Inclusion Criteria as the left • Refuse to give informed consent
JO	• Participants having not been given an anti-obesity medication prescription or received an obesity diagnosis prior to this yearly physical examination	• Being diagnosed as secondary hypertension, heart, kidney, liver, or other endocrine diseases, severe chronic diabetic complications, any recent acute illness, refusal to give informed consent
JH	• Individuals having not had their hypertension identified or treated with any anti-hypertension medications prior to this annual physical check	
OH	• Subjects having not been given a diagnosis of obesity or hypertension and not received any treatment for those conditions until this annual physical examination	

NH group, the normal healthy group; JO group, the newly diagnosed untreated just obesity group; JH group, the newly diagnosed untreated justhypertension group; OH group, the newly diagnosed untreated obesityhypertension group

This study followed the Declaration of Helsinki and received approval from the Hangzhou Center for Disease Control and Prevention’s ethical committee. All participants agreed to join this study and signed a free and informed consent form [30–34].

### Measurement protocol

The participants’ weight, height, waist circumference (WC) were measured according to standard protocols as previously described [30–34]. Before the measurement, the participants removed their shoes, heavy clothing, belts, and any other items that could interfere with the waist circumference (WC) measurement [35]. The WC was measured at the midpoint between the lower rib margin and the iliac crest while the participants gently exhaled [36].

General Obesity was defined as BMI ≥ 28 kg/m^2^ and central obesity was defined as WC ≥ 85 cm for males and ≥ 80 cm for females according to the definition created by the Chinese Obesity Working Group [30–34]. In the present study, BMI was used to identify obesity, i.e., the general obesity was used to define the subject groups [37, 38]. The visceral adiposity index (VAI) was calculated using a formula that incorporates WC, BMI, triglyceride (TG), and high-density lipoprotein cholesterol (HDLC) levels, i.e.,VAImen = [WC/(39.68 + 1.88 × BMI)] × (TG/1.03) × (1.31/HDLC); VAIwomen = [WC/(36.58 + 1.89 × BMI)] × (TG/0.81) × (1.52/HDLC) [39, 40].

The blood pressure is considered a good indicator of the status of the cardiovascular system. The blood pressure can be measured by direct intraarterial and indirect sphygmomanometer methods [41]. In this study, all participants had blood pressure measured using indirect method with electronic sphygmomanometer (HEM-4021, Omron, Kyoto, Japan) [42, 43]. Blood pressure was measured after at least 10 minutes of rest in a seated position using a standard protocol [44]. And the subject should avoid smoking, drinking caffeine, or exercising within 30 minutes of the test [45]. The inflatable cuff of the sphygmomanometer wrapped around the right upper arm at the same level as the heart and the arm was supported on a flat surface [46, 47]. Three measurements were taken with 5 to 15 minutes intervals and the average of three such systolic and diastolic blood pressure readings was taken as the examination blood pressure for the further analysis [44].

Hypertension was defined according to the recommendation of the Joint National Committee on Detection, Evaluation and Treatment of High Blood Pressure and the World Health Organization–International Society of Hypertension as a systolic blood pressure of 140 mmHg or higher and/or a diastolic blood pressure of 90 mmHg or higher at each of the three appointments [44, 48, 49].

### Serum adiponectin level and other metabolic parameters

The routine clinical laboratory tests were carried out on the subjects’ blood and urine samples. Blood samples were collected after an overnight fast and frozen for later analysis. Serum adiponectin level was quantified using a commercially available ELISA kit that measures the total adiponectin concentration (including all isoforms) [31, 50]. According to the anamnesis and other clinical records, general demographic data, such as age and gender, as well as some biochemical parameters (see Table 2), were obtained [30–34].

**Table 2:** Anthropometric and biochemical characteristics of the four groups

Items	JO	OH	JH	NH
	Median (P25–P75)/number	Median (P25–P75)/number	Median (P25–P75)/number	Median (P25–P75)/number
Sex, male/female	44/11	35/7	25/5	15/11
Adiponectin (ng/mL)	1808.4(1356.2–2270.4)*	2012.1(1463.4–2679.4)	1855.3(1549–2869.8)	2301.4(1703.3–2953.6)
VAI	4.7(2.9–6.9)*	4(2.6–6.7)*	3.7(2.8–5.6)	2.8(1.9–3.7)
Age	36(30–44)	43(28.8–56)	49.5(33.8–54)	35.5(31–40.5)
BMI (kg/m^2^)	29.1(28.6–29.8)	29.7(28.6–31)	23.7(22.3–26.1)	22(20.6–23.8)
Body weight	85(76–91)	86.5(80.3–90.8)	70(61.5–77.5)	63.75(55.9–69.6)
WC (cm)	98(93–101)	100(96–103)	90.5(83.8–96.5)	83.5(79.8–87)
SB (mmHg)	130(120–136)	148(141–154.5)	145(142–149)	115.5(106.8–125.3)
DB (mmHg)	79(71–85)	91(86–95)	91(86–97)	70(67–74.3)
ALB (g/L)	46.3(44.7–48.2)	46.5(44.1–47.8)	46.4(45.2–47.9)	48.2(46.5–49.6)
ALT (U/L)	32(23–50)	25(19.8–38.3)	22.5(14–35.3)	14(11–20)
LDLC (mmol/L)	102(84–123)	106.5(91.5–128.3)	108(91.5–121.5)	89(78.8–109)
TG (mg/dl)	137(94–236)	143.5(89–231.3)	137(99–213)	102.5(68–129.8)
HDLC (mg/dl)	44(39–52)	49(44–55.3)	50.5(43.8–66.5)	55(48.8–63.8)
FA (mmol/L)	1.5(1.4–1.7)	1.6(1.5–1.8)	1.5(1.4–1.8)	1.5(1.4–1.6)
SCR (μmol/L)	86(79–90)	86(77–92.3)	83(76.8–90.5)	85.5(68–90.3)
IB (μmol/L)	7.5(5.9–8.7)	7.6(6.3–10.1)	7.5(5.8–8.4)	8.6(6.3–11.3)
ALP (U/L)	64(55–75)	65(54.8–78.8)	68.5(57.8–80)	52.5(46–69.5)
CB (μmol/L)	3.5(2.3–4.4)	3.6(2.8–4.5)	3.5(2.9–4.4)	4.3(3.2–4.9)
UREA (mmol/L)	4.7(4–5.8)	4.9(4.2–6)	5.2(4.9–6.3)	4.4(3.8–5.1)
Ua (μmol/L)	354(286–413)	360.5(303.5–415.8)	348.5(281.3–388.5)	270(246–355.3)
GLU (mmol/L)	5.1(4.7–5.4)	5.1(4.7–5.8)	5(4.7–5.5)	5.1(4.7–5.3)
TC (mg/dl)	190(171–221)	202.5(181–235.5)	203.5(181.3–225.5)	192.5(170.8–206.8)
TB (μmol/L)	10.9(8.6–12.9)	11.2(9.4–14)	10.7(8.8–12.4)	12.5(8.9–16.7)
TP (g/L)	77.5(74.8–80.1)	77.3(75.2–79.8)	75.4(73.3–77.7)	75.8(72.4–78.4)
GGT (U/L)	33(23–44)	32(20.5–44.3)	25(16.8–42.3)	14(10–18.5)

Data about male/female are presented with the number, and other data are expressed as Median (interquartile range), *: *p* < 0.05 (significant), compared with NH after adjustment for gender and age, *SB* systolic blood pressure, *DB* diastolic blood pressure, *ALB* Albumin, *ALT* Alanine aminotransferase, *LDLC* Low-density lipoprotein cholesterol, *TG* Triglyceride, *HDLC* High-density lipoprotein cholesterol, *FA* Fructosamine, *SCR* serum creatinine, *IB* Indirect bilirubin, *ALP* Alkaline phosphatase, *CB* Conjugated bilirubin, *UREA* Urea, *Ua *Uric acid, *GLU* fasting blood glucose, *TC* Total cholesterol, *TB* Total bilirubin, *TP* Total protein, *BMI* body mass index, *WC* waist circumference, *GGT* Gamma-glutamyltransferase, *VAI* The visceral adiposity index

## Statistical analysis

R software (https://www.r-project.org) and SPSS for Windows V22.0 (IBM Corp., Armonk, NY) were used to perform the statistical analyses. The median and the interquartile range (P25, P75) were reported as descriptive statistics. Pearson’s test were applied to assess the associations between groups when the data (or transformed data) met the normality and equal variance assumptions; otherwise, the Mann–Whitney rank sum test and Spearman’s correlation test were used. The tested index among the four subgroups were compared using the rank-sum or T-test. Logistic regression, partial correlation or Fisher exact test were also employed when appropriate. The significance level at 0.05 was set for two-sided tests. Multiple regression models were fitted to study the independent association of the study variables with serum adiponectin, and SB (or DB).

## Results

### Characteristics

The table 2 shows the baseline characteristics of the subjects in the four groups, including their general and anthropometric data and biochemical parameters. The table presents the median, the quartiles (P25, P75), and the number of the following variables: GGT, ALB, ALT, LDLC, TG, HDLC, FA, SCR, IB, ALP, CB, GLU, TC, TB, TP, Ua, UREA, age, gender, and WC, BMI, SB, and DB. The full names of these indicators can be found in the notes below the Table 2.

### The relationship between VAI and serum concentrations of adiponectin

The median (P25, P75) of VAI and serum adiponectin levels of the four groups are shown in Table 2.

After adjustment for gender and age, analysis of covariance (ANCOVA) revealed that the serum adiponectin level in the NH group was significantly higher than in the JO groups (*P* = 0.036). No significant differences of the serum adiponectin between other two groups. The order of serum adiponectin levels among the groups was NH > OH > JH > JO.

The difference of VAI between NH group and JO group (or the OH group) is statistically significant (*P* < 0.05) after adjusting for gender and age. The VAI of NH group is the smallest among the four subgroups. The difference of VAI between NH group and JO group is nearly statistically significant (*P* = 0.071). The *P*-value for the comparison of VAI between JO and OH groups was 0.376, which means that the difference was not statistically significant after adjustment for gender and age.

And, after adjustment for gender and age, partial correlation analysis revealed that serum adiponectin had a negative association with VAI that was trending towards significance (*P =* 0.055, *r* = − 0.157). All the data were log-transformed to follow a normal distribution before analysis.

### Multiple regression models for the independent association of the study variables with serum adiponectin

A linear regression model towards serum adiponectin is shown in Table 3 (*R*^2^ = 0.314). Gender, ALB, ALT and HDLC were found to be independent determinants. The other variables did not reach significance in the model (*P* > 0.1). This model was responsible for 31.4% of the original variation of the serum adiponectin.

**Table 3:** Multiple linear regression model for the predictors of serum adiponectin in the total study population (*n* = 153)

Variables	Unstd. B	SE(B)	std.β	*P*-value
Intercept	3369.369	2326.412	---	0.150
gender	708.560	247.389	0.323	0.005**
ALT	2.734	2.265	0.102	0.046*
HDLC	16.896	8.521	0.228	0.049*
ALB	−106.504	42.371	−0.269	0.013*

*: *p* < 0.05 (significant), **: *p* < 0.01 (highly significant), the other variables did not reach significance in the model (*p* > 0.1), “Unstd. B” in the table refers to the unstandardized coefficients, “SE(B)” refers to the standard error for the unstandardized coefficients,“std.β” refers to standardized coefficients, *R*^2^ = 0.314, *HDLC* High-density lipoprotein cholesterol, *ALB* Albumin, *ALT* Alanine aminotransferase, Ua, Uric acid

### The difference of serum adiponectin levels between genders in the four group

Table 4 shows the comparisons of the serum adiponectin levels between genders in the four groups of subjects: JO, OH, JH, and NH. The table presents the median and the interquartile range of the serum adiponectin levels for each group and gender. The table also reports the *P*-values of the differences between males and females within each group. The results indicate that after the age was controlled, the serum adiponectin levels were significantly higher in females than in males in the OH and NH groups, but not in the JO and JH groups. This suggests that there is a sexual difference in the serum adiponectin levels, which may be influenced by the presence or absence of obesity and hypertension.

**Table 4:** Comparisons of the serum adiponectin (ng/mL) between genders in the four groups

Group	*P*-value	Gender	adiponectin (ng/mL)
			median (p25-p75)
JO	0.512	male	1742.3(1347.9–2171.5)
		female	2096.1(1526.9–2868.6)
OH	0.009**	male	1734(1384.3–2284.2)
		female	2857.6(2298.5–3875.4)
JH	0.877	male	1866.5(1537.9–2915.3)
		female	1643.5(1184.6–4213.5)
NH	0.001**	male	1961.6(1593.5–2366.1)
		female	2943.7(2716.6–3938.4)

**: *p* < 0.01 (highly significant);NH, normal healthy group; JH, newly;diagnosed untreated just-hypertension group; JO, newly diagnosed untreated;just-obesity group; OH, newly diagnosed untreated obesity-hypertension group

### Multiple regression models for the independent association of the study variables with SB and DB

The linear regression models towards SB and DB are shown in Table 5 (*R*^2^ = 0.333) and Table 6(*R*^2^ = 0.230), respectively. For SB, age and HDLC were found to be independent determinants, and ALB was borderline significant. ALP barely escaped statistical significance The other variables did not reach significance in the model (*P* > 0.08). This model was responsible for 33.3% of the original variation of the SB serum adiponectin. For DB, ALP was found to be the independent determinant, and age had a considerable trend toward significance.

**Table 5:** Multiple linear regression model for the predictors of SB in the total study population (*n* = 153)

Variables	Unstd. B	SE(B)	std.β	*P*-value
Intercept	90.990	41.699	---	0.031*
age	0.291	0.135	0.202	0.032*
HDLC	0.305	0.134	0.222	0.025*
ALB	−10.500	0.768	−0.204	0.053
ALP	0.144	0.081	0.137	0.078
adiponectin	−0.001	0.002	−0.079	0.357

*: *p* < 0.05 (significant), **: *p* < 0.01 (highly significant); the other variables did not reach significance in the model (*p* > 0.08); “Unstd. B” in the table refers to the unstandardized coefficients; “SE(B)” refers to the standard error for the unstandardized coefficients;“std.β” refers to standardized coefficients; *R*^2^ = 0.333; HDLC, High-density lipoprotein cholesterol; ALB, Albumin; ALP, alkaline phosphatase, IB, alkaline phosphatase

**Table 6:** Multiple linear regression model for the predictors of DB in the total study population (*n* = 153)

Variables	Unstd. B	SE(B)	std.β	*P*-value
Intercept	30.660	31.157	---	0.327
ALP	0.141	0.061	0.192	0.022*
age	0.180	0.101	0.179	0.076
adiponectin	0.000	0.001	0.031	0.737

*: *p* < 0.05 (significant); the other variables did not reach significance in the model (*p* > 0.08); “Unstd. B” in the table refers to the unstandardized coefficients; “SE(B)” refers to the standard error for the unstandardized coefficients;“std.β” refers to standardized coefficients; *R*^2^ = 0.230; ALP, alkaline phosphatase

## Discussion

Adiponectin is primarily produced and released by energy-storing cells in adipose tissue (body fat) called adipocytes [51–55]. Yet, other tissues and other types of cells can produce adiponectin, including osteoblasts, myocytes, heart muscle cells, liver parenchyma cells,endothelial cells and placental tissue [51–61]. Adiponectin is known as the most abundant adipokine and its paradoxical elevation in some disorders is receiving more attention [62–64]. Adeno-viral over-expression of adiponectin in genetically obese KKAy/a mice with obesity-related hypertension resulted in reduced blood pressure [8]. However, normal wild-type mice and mice with specific genetic backgrounds may exhibit different mechanisms for obesity-related hypertension and may also show varying roles of adiponectin in the context of this condition [65, 66].

This study investigated the role of adiponectin in obesity-hypertension by measuring its serum levels and correlations with various parameters in four groups of Chinese adults: NH, JO, JH, and OH.

Though adiponectin is mainly secreted by adipose tissue (AT), its circulating concentration is decreased in obesity [51–55]. The results in this study also demonstrated that the serum adiponectin level in the NH group is significantly higher than in the JO group. AT can broadly be divided into two types with distinct roles: white AT (WAT) and brown AT (BAT) [56, 67]. BAT has smaller lipid droplets and more blood vessels than WAT, resulting in a darker, brown macroscopic look [68, 69]. And beige adipose tissue, a type of inducible BAT, intermingles with WAT depots and occurs in response to cold exposure and pharmacological modulation of WAT [70–72]. This phenomenon of decreased serum adiponectin level in obese people might be attributed to fat cell dysfunction and/or hypermethylation of the adiponectin gene in morbid obesity [73, 74]. Some previous literature also shows that the adiponectin paradox, which refers to the paradoxical elevation of adiponectin in some disorders, is more evident in non-obese than in obese patients with diabetic microvascular complications [75]. The authors suggested that the paradoxical elevation of adiponectin in vascular damage might be a compensatory response, and that the responsive upregulation might be insufficient in obese patients [75]. Previous studies revealed that the quality of adipose tissue is largely compromised in patients with severe cardiovascular disorders [76, 77]. Additionally, during caloric restriction (CR), increased circulating adiponectin is observed to come from bone marrow adipose tissue (MAT) [78, 79].

And, it was found in this study that serum adiponectin levels negatively correlated with VAI. VAI is a mathematical formula that estimates the amount and function of visceral adipose tissue (VAT), which is the fat that surrounds the internal organs in the abdomen [80–83]. WAT can be divided into two major depots: VAT and subcutaneous adipose tissue (SAT). SAT is located under the skin. The distribution of WAT affects the production and function of adiponectin [84, 85]. Generally, VAT is associated with lower adiponectin levels and higher risk of metabolic and cardiovascular complications, such as diabetes, hypertension, and atherosclerosis [86–88]. This is because VAT is more prone to inflammation, insulin resistance, and lipolysis, which can impair adiponectin secretion and action [86–88]. On the other hand, SAT is mainly considered to be associated with higher adiponectin levels and lower risk of metabolic diseases [89, 90], though with the oppsite results exsiting [91]. This is because SAT is more responsive to insulin, has anti-inflammatory properties, and can store excess lipids away from harmful sites [92, 93]. However, not all SAT depots are equal. Fat on the lower extremities, such as the legs, is more beneficial than fat on the upper body, such as the abdomen [93, 94]. This is because lower-body fat has higher adiponectin expression and secretion, and can protect against the adverse effects of VAT [84, 94, 95]. Therefore, the role of WAT distribution in adiponectin levels and metabolic health is complex and depends on the location, amount, and function of different fat depots [91, 93, 96, 97]. A better understanding of the molecular mechanisms that regulate adipocyte differentiation and adiponectin secretion in different WAT subtypes may lead to new therapeutic strategies for obesity and its related complications [91, 93, 96, 97]. VAI takes into account waist circumference, body mass index, triglycerides, and HDLC levels, and it is different for men and women [80–82]. VAI has been shown to be a reliable indicator of visceral fat dysfunction and cardiometabolic risk [98]. VAI seems to represent a better predictive tool than common clinical parameters for metabolic disorders in Chinese and Caucasian samples [82, 99, 100].

And, in this study, we found that the NH group had a much lower VAI than almost all other groups (JO, JH, OH). The JH group also had a lower VAI than the OH group. Previous study reported weekend warrior activity patterns (WWs) and regular activity patterns offer the same benefits for reducing the VAI [101]. This suggests that the NH group and JH group might have higher level of physical activity and a larger proportion of muscle mass in their body weight than the other two groups. Aditionally, the serum adiponectin levels negatively correlated with VAI in this study. These results reflect that serum adiponectin levels positively correlated with physical activity and muscle mass proportion of the body. It has been shown that physical exercise can reduce fat mass [76], especially the accumulation of VAT [102–104], and lead to hypersecretion of adiponectin [102–104], which results in increased adiponectin production in adipose tissue and enhanced concentration of adiponectin in the blood [105–108]. Our study result accords with the phenomena discovered in those previous literature.

In this study, the comparison of VAI between JO and OH groups revealed no significant difference. This indicates that the VAI, a marker of visceral adiposity and cardiometabolic risk, was similar between the two groups. However, the increase of the serum adiponectin level in OH compared to JO may seem paradoxical, as adiponectin is generally considered to have anti-obesity and anti-hypertensive effects [9, 109]. Nevertheless, the result was in line with the phenomenon called “adiponectin paradox” in which adiponectin levels are elevated in some pathological conditions, such as cardiovascular disease, diabetes, and chronic kidney disease [76]. This may reflect a compensatory response of the body to maintain metabolic homeostasis and protect against further damage [9, 110]. Moreover, adiponectin levels may be influenced by other factors, such as gender, albumin, alanine aminotransferase, and high-density lipoprotein cholesterol, as we showed in the multiple regression analysis. And the interaction between adiponectin and other adipokines, such as leptin, resistin, and visfatin, should be taken into account [111, 112]. In addition to white adipokines (adipokines produced by WAT), the brown adipokines, adipokines present in brown adipose tissue, such as fibroblast growth factor 21 (FGF21), interleukin-6 (IL-6), neuregulin-4 (NRG4), insulin-like growth factor-1 (IGF-1), and tumor necrosis factor-alpha (TNF-α), may also play a role in the process known as the ‘adiponectin paradox [112, 113]. And these adipokines also have tortuous relationship with hypertension. As shown in previous studies, leptin’s effect on Trpm7 (TRP [transient receptor potential] melastatin 7) expression, via epigenetic changes and the pSTAT3(phosphorylated signal transducer and activator of transcription 3)-JAK2(Janus kinase 2) pathway, contributes to obesity-associated hypertension, but leptin’s role is additive, not essential, in the complex regulatory network leading to hypertension in obesity [33, 114–116]. Depending on the context, resistin appears to have both protective and detrimental effects to obesity-related hypertension, highlighting the complexity of resistin’s role in obesity and hypertension [30]. And, IL-6 may have diverse effects in the pathogenesis of obesity and hypertension, depending on the presence or absence of these conditions [34]. Therefore, the role of adiponectin in obesity-hypertension should be considered in the context of the systemic signaling axis and not in isolation [117].

Next, with the multiple regression model, it was found serum adiponectin level was significantly higer in females than in males (with std.β = 0.323). This result is in line with many previously studies, which demonstrate that adiponectin levels are generally higher in females than in males, especially in adulthood [118, 119]. This may be partly explained by the effects of sex hormones, such as testosterone and estrogen, on adiponectin production and secretion [119, 120]. Testosterone has been shown to suppress adiponectin expression in fat cells, whereas estrogen has been shown to stimulate it [120]. Therefore, during puberty, when sex hormones increase, adiponectin levels tend to decline in males and rise in females [119, 121]. However, the sex differences in adiponectin levels are not consistent across different ethnic groups and obesity levels. For example, some studies have found that Hispanic females have lower adiponectin levels than non-Hispanic white females, and that this difference is more pronounced in obese individuals [122–124]. And dietary patterns may also affect adiponectin levels and the sex differences in this hormone [125–128]. Healthy dietary patterns rich in fruits, vegetables, whole grains, fish, nuts, and unsaturated fats are linked to higher adiponectin levels, likely due to their bioactive compounds that promote adipose tissue function and insulin sensitivity [128–131]. Conversely, unhealthy diets high in red and processed meats, refined grains, sweets, fast foods, and saturated/trans fats correlate with lower adiponectin levels, potentially due to inflammation, oxidative stress, and insulin resistance that hinder adiponectin secretion and activity [128–131]. And a negative association between the Dietary Approaches to Stop Hypertension (DASH) diet index and VAI was reported among older Americans [132].

And the interaction between diet and genetics may modulate adiponectin levels and metabolic outcomes [133]. For eample, ADIPOQ variants conferred more metabolic risks in healthy dietary patterns than in adverse dietary patterns [133, 134]. And some literature indicated that moderate amounts of ethanol-containing beverages increased adiponectin concentrations, and sex-specific effects might depend on type of beverage consumed [135]. This may suggest that other factors, such as genetic, environmental, or lifestyle factors, may also influence adiponectin levels and modify the sex differences [118, 136–138]. In this study, blood samples were obtained after a 12-hour of fasting, which can avoid the short-term effects of the diet. And, after the age was controlled, the serum adiponectin levels were significantly higher in females than in males in the OH and NH groups, but not in the JO and JH groups. This suggests that there is a sexual difference in the serum adiponectin levels, which may be influenced by the presence or absence of obesity and hypertension [139].

According to vast majority of reports, adiponectin has been exhibited to have protective effect on vascular functions and thus has negative relationship with blood pressure, against incidence of obesity-hypertension [21, 140, 141]. But the contrary opinion holds that, in different subject groups, the relationships between the adiponectin and blood pressure are various, with positive and non-correlation existing [24, 142–144]. The preclinical expriments, longitudinal studies and clinical trials have reported inconsistent outcomes regarding the antihypertensive effects of adiponectin [11, 13, 25–29, 145]. In this study, using the multiple regression model, it was found that adiponectin was not associated with blood pressure (SB and DB) after adjustment for other risk factors (the anthropometric and biochemical characteristics mentioned in Table 2 ) used in multiple linear regression models. Age and HDLC were found to be independently positively correlate with SB, and ALB was at the edge of significance. ALP were found to be independently positively correlate with DB, and age has a considerable trend toward significance.

This study results revealed that ALB independently negatively and ALT, HDLC, and gender positively associate with serum level of adiponectin. These results were in line with many previous research outcomes. Serum adiponectin levels have been recorded to display a sexual difference, being higher in females than in males [118, 146–148], and were observed having inverse correlation with circulating *albumin* [120, 149, 150]. And recently, higher plasma adiponectin abundance was discovered in albumin knockout (KO) mice [151].

The associations between adiponectin and ALT have been shown to be different. Many research detailed the opposite connections [152–155], however positive and non-connections are likewise existing [156, 157]. And adiponectin knockout could ablate the significant rise in serum ALT elicited by high-fat diet feeding in mice [158].

Consistent with our study results, the serum HDLC has been reported to be independently positively associated with serum adiponectin in almost all the previous studies [159–165]. Adiponectin could upregulate ATP-binding cassette transporter A1 and G1 expression and hepatic apo-AI (Apolipoprotein A-I), reduce lipid accumulation, and efficiently promote nascent HDLC formation [166–171]. Lower adiponectin (ADIPOQ) gene expression are associated with diabetic dyslipidemia [172, 173].

Accounting for the mentioned above, in this mixed subjects of four Chinese subgroups, adiponectin did not have the dose-response conjunction with the blood pressure after adjustment for potential confounding factors, which was little illustrated in earlier investigations [11]. These conflicting results in different studies may be mostly due to the diversity of subjects included [24]. The direct relationship between adiponectin and hypertension (or blood pressure) may be influenced by many factors, among which the liver and kidney dysfunctions are often mentioned. Disorders of kidney or liver can lead to the elevation of the blood pressure and incident of hypertension [174, 175]. Meanwhile, the impaired liver function can affect adiponectin’s degradation in the liver, and the kidney dysfunctions can reduce renal clearance of adiponectin [174–176]. High serum ALT is known to reflect the situation of severe liver disease. In addition, in this study, ALP was found to independently positively correlate with DB and nearly with SB. High levels of ALP in the blood may indicate liver disease or certain bone disorders. Furthermore, in this study, ALB was found to have a negative association with SB that was trending towards significance. Low albumin levels in the blood are known to indicate serious liver and kidney problems.

Another explanation for the increase of adiponectin in individuals with risk towards mortality is that is a failing attempt of the body to do the protection [177], as pathological adiponectin resistance develops with its receptors down regulation in metabolically active organs including the adipose tissue, the heart, the liver and the vasculature [172, 173, 178]. Adiponectin exerts its both beneficial and detrimental effects via normal or impaired signaling through its receptors: Adiponectin receptor 1 and 2 (AdipoR1 and AdipoR2), T-cadherin and calreticulin [1]. Adiponectin exists in various isoforms in the circulation, including trimer (~ 67 kDa; low molecular weight, LMW), hexamer (136 kDa; middle molecular weight, MMW), 12-32mer (> 300 kDa, high molecular weight, HMW), and globular forms [76, 179, 180]. Different adiponectin isoforms bind to different receptors, resulting in various functions. Globular adiponectin preferentially binds to AdipoR1, influencing muscle cells [56]. T-cadherin, a non-transmembrane binding protein, is a key partner for HMW adiponectin, potentially accumulating it in heart, vascular endothelium, and muscle, with its downstream effects being studied [181–183]. Αdiponectin may bind to calreticulin on macrophage surfaces and other cells [184, 185]. AdipoR2 binds full-length adiponectin and is predominantly expressed in the liver [186]. However, the relative contributions of different adiponectin isoforms to obesity and hypertension are not fully understood and may vary depending on the population and the disease stage [180].

In general, our research investigated the role of adiponectin in obesity-hypertension using a case-control study with four groups of Chinese adults: normal healthy, just-obesity, just-hypertension, and obesity-hypertension. This design allows for a comprehensive comparison of the serum adiponectin levels and their associations with various anthropometric and biochemical parameters among different subgroups of obesity and hypertension. Thus, this research provided more clues for distinguishing the antinomy of adiponectin in obesity-hypertension by analyzing the correlations between adiponectin and visceral adiposity index, gender, serum albumin (ALB),alanine aminotransferase (ALT) and high-density lipoprotein cholesterol (HDLC), blood pressure, and other indicators. This research also indicated some factors that influence the sex differences in adiponectin. Next, the results of this research suggested that adiponectin may not always have a direct relationship with blood pressure. The importance of adiponectin must be viewed within the framework of the body’s key signaling pathways that regulate metabolic equilibrium in the face of obesity and cardiovascular disorders [187], such as the modulation by other adipokines (leptin, resistin, and visfatin, as well as brown adipokines, such as FGF21, IL-6, NRG4, IGF-1, and TNF-α). This research also indicated the potential mechanisms of adiponectin in regulating carbohydrate and lipid metabolism, inflammation, and vascular function. Furthermore, Different explanations for the adiponectin paradox have been discussed including adiponectin resistance, compensatory effects of adiponectin to subclinical pathologies, impaired renal function and decreased hepatic clearance of adiponectin [188–190]. And this research revealed that adiponectin’s role in obesity-hypertension is complex and depends on the systemic metabolic homeostasis signaling axis. And in the end, the results of this research indicated that in the context of obesity-related hypertension, compensatory effects, adiponectin resistance, and decreased adiponectin clearance due to impaired renal and hepatic function occur simultaneously, contributing to the “adiponectin paradox”.

Some limitations and strengths of our study should be addressed. At first, our results need to be interpreted in the context of a few limitations. One limitation is that the cross-sectional design and small sample size of our study and the subjects restricted to Chinese adults may limit the generalizability and causal inference of our findings. Therefore, Our results should be interpreted with caution and should be confirmed by larger, longitudinal studies across various genetic populations in the future. Another limitation is that we did not measure the adiponectin isoforms, which could have provided more nuanced insights into the role of adiponectin in obesity-hypertension. Previous studies have reported conflicting results on the associations of adiponectin isoforms and other adipokines (WAT or BAT adipokines) with blood pressure and metabolic parameters in different populations [56, 112, 179, 180]. Therefore, further studies are needed to investigate the differential effects of adiponectin isoforms on obesity-hypertension and to explore the potential mechanisms underlying the adiponectin paradox. Third limitation is that while the present study attempts to control for various confounders, there are still potential variables that might influence the results, such as dietary patterns, genetic predispositions, and environmental factors. We did not have comprehensive dietary data and we did not delve into the relationship between adiponectin and other adipokines (other BAT adipokines or other WAT adipokines). Finally, the VAI is not a direct and accurate measure of physical activity and that it may not capture the intensity, duration, and type of physical activity that the participants engaged in.

Notwithstanding these limitations, the multiple strengths of our study should be kept in mind. Firstly, the chosen population in the present study allowed us to investigate the role of adiponectin in obesity-hypertension axis in a relatively homogeneous and early-stage group, without the confounding effects of medication or other comorbidities. Secondly, to minimize the effects of diet, blood samples were obtained after a 12-hour fast in the present study to avoid the short-term effects of recent dietary intake. Thirdly, we have controlled for potential confounding factors, such as age, gender, and other biochemical indicators. And the use of multiple regression models adds rigor to the findings, offering insights into the independent determinants of serum adiponectin levels. Fourthly, the Visceral Adiposity Index (VAI), utilized in this study, serves as a comprehensive indicator of physical activity levels [98, 101]. It inversely correlates with physical activity and positively correlates with visceral fat dysfunction and cardiometabolic risk, making it a superior predictive tool for metabolic disorders in both Chinese and Caucasian populations [82, 99, 100]. Fifthly,we believe that our findings may have some relevance and applicability to other populations and settings because of the consistent with some previous studies that have reported no directly associations between adiponectin and blood pressure in different ethnic groups and regions, such as African Americans, Europeans, and Japanese [27, 191, 192]. These studies suggest that the “adiponectin paradox” may be a common phenomenon in obesity-hypertension, regardless of the population characteristics.

In summary, our study findings provide new insights into the “adiponectin paradox”. Adiponectin’s role in obesity-hypertension is multifaceted and is influenced by the systemic metabolic homeostasis signaling axis. In obesity-related hypertension, compensatory effects, adiponectin resistance, and reduced adiponectin clearance from impaired kidneys and liver all contribute to the “adiponectin paradox”.

And to further elucidate the role of adiponectin in obesity-hypertension, more studies are needed to investigate the adiponectin isoforms, the adiponectin receptors, and the signaling pathways involved in different tissues and organs. Moreover, the interactions between adiponectin and other adipokines, hormones, and inflammatory mediators should be explored in the context of obesity-hypertension. Additionally, the genetic, environmental, and lifestyle factors (such as dietary assessments including alcohol intake and direct measurement of physical activity) that influence adiponectin levels and function should be considered [173]. Finally, the therapeutic potential of adiponectin or its analogues for obesity-hypertension should be evaluated in clinical trials.

## Data Availability

The datasets used and/or analysed during the current study available from the corresponding author on reasonable request.

## Abbreviations

WC: Waist circumference
BMI: Body mass index
SB: Systolic blood pressure
DB: Diastolic blood pressure
HMW: High molecular weight
eNOS: Endothelial nitric oxide synthase
NO: Nitric oxide
AMPK: Adenosine 5’-monophosphate (AMP)-activated protein kinase
COX-2: cyclooxygenase-2
PGI2: Prostaglandin I2
Akt: Protein kinase B
GGT: Gamma-glutamyltransferase
ALB: Albumin
ALT: Alanine aminotransferase
LDLC: Low density lipoprotein cholesterol
TG: Triglyceride
HDLC: High density lipoprotein cholesterol
FA: Fructosamine
SCR: Serum creatinine
IB: Indirect bilirubin
ALP: Alkaline phosphatase
CB: Conjugated bilirubin
UREA: Urea
Ua: uric acid
FBG: Fasting blood glucose
TC: Total cholesterol
TB: Total bilirubin
TP: Total protein
VAI: Visceral adiposity index
AT: Adipose tissue
WAT: White adipose tissue
BAT: Brown adipose tissue
MAT: Bone marrow adipose tissue
CR: Caloric restriction
VAT: Visceral adipose tissue
SAT: Subcutaneous adipose tissue
FGF21: Fibroblast growth factor 21
IL-6: Interleukin-6
NRG4: Neuregulin-4
IGF-1: Insulin-like growth factor-1
TNF-α: Tumor necrosis factor-alpha
Trpm7: TRP [transient receptor potential] melastatin 7
JAK2: Janus kinase 2
pSTAT3: Phosphorylated signal transducer and activator of transcription 3
apo-AI: Apolipoprotein A-I
LMW: Low molecular weight
MMW: Middle molecular weight
AdipoR1: Adiponectin receptor 1
AdipoR2: Adiponectin receptor 2
DASH: Dietary approaches to stop hypertension
WWs: Weekend warrior activity patterns
ANCOVA: Analysis of covariance
ABCA1: ATP-binding cassette transporter A1
ABCG1: ATP-binding cassette transporter G1
KO: Knockout
